# UBE2C serves as a prognosis biomarker of uterine corpus endometrial carcinoma via promoting tumor migration and invasion

**DOI:** 10.1038/s41598-023-44189-1

**Published:** 2023-10-06

**Authors:** Sijia Ma, Qian Chen, Xu Li, Jing Fu, Le Zhao

**Affiliations:** 1 Center for Translational Medicine, The First Affiliated Hospital of Xi’an Jiaotong University, Xi’an, Shaanxi 710061 People’s Republic of China; 2 Department of Obstetrics and Gynecology, The First Affiliated Hospital of Xi’an Jiaotong University, Xi’an, Shaanxi 710061 People’s Republic of China; 3 Key Laboratory for Tumor Precision Medicine of Shaanxi Province, The First Affiliated Hospital of Xi’an Jiaotong University, Xi’an, Shaanxi 710061 People’s Republic of China; 4 Department of Obstetrics and Gynecology, The Second Affiliated Hospital of Xi’an Jiaotong University, Xi’an, Shaanxi 710061 People’s Republic of China

**Keywords:** Biophysics, Cancer, Cell biology, Computational biology and bioinformatics, Genetics

## Abstract

The biological functions of ubiquitin-conjugating enzymes E2 (UBE2) family members in uterine corpus endometrial carcinoma (UCEC) remains unclear. Our study aimed to systematically analyze the expression patterns, prognostic value, biological functions and molecular regulatory mechanisms of UBE2 family in UCEC. Among nine screened UBE2 family members associated with UCEC, UBE2C was the most significantly overexpressed gene with poor prognosis. High expression levels of UBE2C in UCEC was correlated with stages, histological subtypes, patient’s menopause status and TP53 mutation. Three molecules (CDC20, PTTG1 and AURKA), were identified as the key co-expression proteins of UBE2C. The generic alterations (mutation, amplification) and DNA hypomethylation might contribute to UBE2C’s high expression in UCEC. Furthermore, in vitro experiments showed that the interference of UBE2C inhibited the migration and invasion of endometrial cancer cells, while partially impact cell proliferation and didn’t impact the expression of epithelial-mesenchymal transition (EMT) markers. Using comprehensive bioinformatics analysis and in vitro experiments, our study provided a novel insight into the oncogenic role of UBE2 family, specifically UBE2C in UCEC. UBE2C might serve as an effective biomarker to predict poor prognosis and a potential therapeutic target in clinical practice.

## Introduction

Uterine corpus endometrial carcinoma (UCEC) is one of the most prevalent and lethal gynecologic malignances, with a global incidence of 1.3% per year from 2007‐2016. In the United States, 66,570 estimated new cases and 12,940 estimated deaths of UCEC have been reported in 2020^1–3^. In China, UCEC is the second most common gynecologic malignancy^4^. The 5-year relative survival rate for all-stage patients is around 81%^5^. However, the prognosis of UCEC patients with distant metastasis remains poor, with as low as 16% of the 5-year relative survival rate^3^. Stagnant survival rate largely reflects a lack of major treatment advances for patients with recurrent and metastatic UCEC^6^. Therefore, it is pivotal to discover novel and effective biomarkers for targeted therapy and prognosis assessment of UCEC.

Ubiquitin (Ub) regulates the stability and biological function of many key hub proteins via the ubiquitin proteasome system (UPS). The aberrant function of the UPS is correlated with many diseases such as malignancy, neurodegeneration, and infectious disease. By a sequential cascade of Ub-activating (E1), Ub-conjugating (E2), and Ub-ligating (E3) enzymes, Ub is added to protein substrates^7–11^. The Ub-conjugating (E2) family members (UBE2s) are imperative in the ubiquitination cascade. Until now, 40 members of UBE2 family have been identified, from UBE2A^12^, UBE2C^13^, UBE2G2^14^, UBE2J1^15^, UBE2S^16^, UBE2T^11^ to UBEV2^17^, and theirs dysregulations impact cell cycle, apoptosis, DNA repair and oncogenic signaling pathways.UBE2s could serve as markers for tumor diagnosis, targeted therapy and prognosis prediction^18^. For example, UBE2C is upregulated and associated with poor survival of UCEC patients^19^, UBE2S is associated with cancer development in non-small cell lung cancer^20^, and the increased expression of UBE2T predicts the poor survival of ovarian cancer patients^11^. However, many of the abovementioned researches are mainly relied on the incomplete bioinformatical analysis or immunofluorescence histochemistry assays, biological function and underlying action mechanism of these UBE2s in UCEC remain to be fully elucidated.

In recent years, many comprehensive public databases have been established, genome, RNA and protein-wide data have been integrated^21^. A large number of biomarkers of cancer and non-cancer diseases have been discovered by data mining^22^. The wide application of comprehensive public databases and computational tools provides an alternative strategy to identify novel biomarkers in a cost-effective way.

In this study, we selected nine gene members of the UBE2 family highly possibly associated with UCEC to investigate and evaluate their expression levels and clinical prognostic values by performing comprehensive bioinformatics analysis. Among the nine UBE2 family members, UBE2C exhibited a significantly strong correlation with UCEC. The association of UBE2C with patient prognosis, pathway enrichment, protein–protein interaction and potential regulatory factors in UCEC were further explored. Our results contributed to the clarification of the role of UBE2C in UCEC.

## Results

### mRNA level and prognosis analysis of UBE2 family members in UCEC

**Figure 1:**
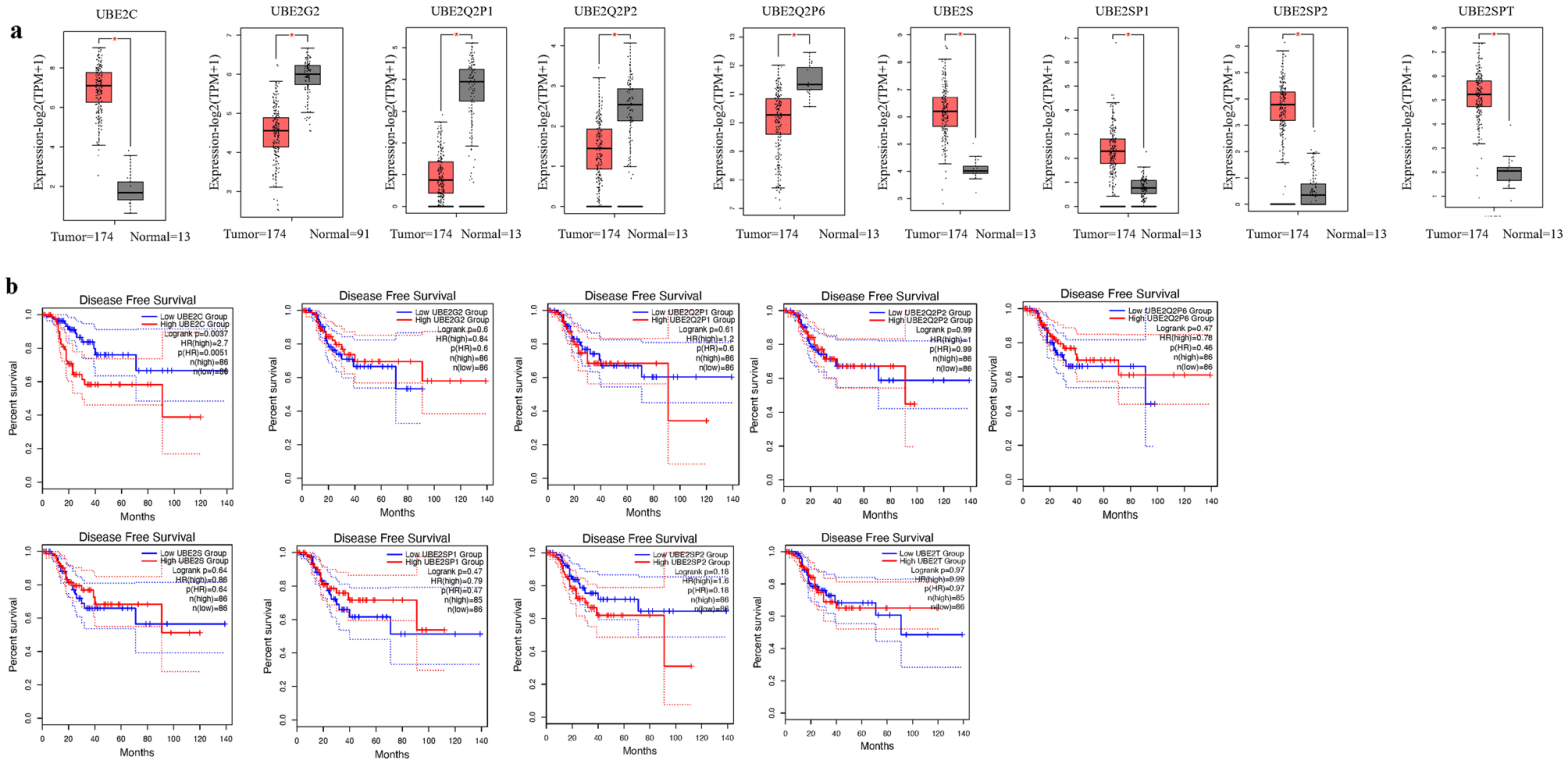
The mRNA expression levels and prognosis analysis of nine UBE2 family genes in UCEC (GEPIA). (**a**) Expression-log2(TPM + 1) of nine UBE2 genes in UCEC compared with normal tissues. The * represent for p-value less than 0.05. (**b**) Disease-free survival of nine UBE2 genes in UCEC.

After comparing the intersection between the common UBE2 family members and the differentially expressed genes in UCEC relative to normal endometrial tissues, nine UBE2 family genes including UBE2C, UBE2G, UBE2G2, UBE2Q2P1, UBE2Q2P2, UBE2Q2P6, UBE2S, UBE2SPA, UBE2SP2, and UBE2T were screened out as having a possible relationship with UCEC (Table 1). Online database, GEPIA was applied to analyze the mRNA levels and prognosis values of these nine UBE2 family members in UCEC compared with normal endometrial tissues. Among the nine significantly differentially expressed genes, five genes (UBE2C, UBE2S, UBE2SP1, UBE2SP2 and UBE2SPT) were upregulated and four genes (UBE2G2, UBE2Q2P1, UBE2Q2P2 and UBE2Q2P6) were down-regulated in UCEC (Fig. 1a). Furthermore, the disease-free survival showed that UBE2C was the only significantly differentially expressed gene to potentially predict patients’ prognosis (Fig. 1b). The higher of UBE2C mRNA, the poorer of the survival. The role and expression regulation mechanism of UBE2C were then investigated in UCEC.

**Table 1:** Nine Screened UBE2 Family genes associated with UCEC.

	Gene ID	Median (Tumor)	Median (Normal)	Log2 (Fold Change)	adjp
UBE2C	ENSG00000175063.16	136.953	0.95	6.145	3.45E − 76
UBE2G2	ENSG00000184787.18	22.605	63.1	− 1.441	2.36E − 21
UBE2Q2P1	ENSG00000189136.8	0.775	14.23	− 3.101	3.57E − 39
UBE2Q2P2	ENSG00000259429.5	1.725	4.82	− 1.095	1.48E − 10
UBE2Q2P6	ENSG00000275695.1	0.07	1.26	− 1.079	8.30E − 28
UBE2S	ENSG00000108106.13	72.627	14.1	2.286	5.78E − 40
UBE2SP1	ENSG00000233966.1	3.89	0.7	1.524	8.93E − 23
UBE2SP2	ENSG00000224126.2	12.82	0.27	3.444	1.45E − 55
UBE2T	ENSG00000077152.9	36.78	2.77	3.325	1.04E − 51

### UBE2C mRNA level in UCEC

**Figure 2:**
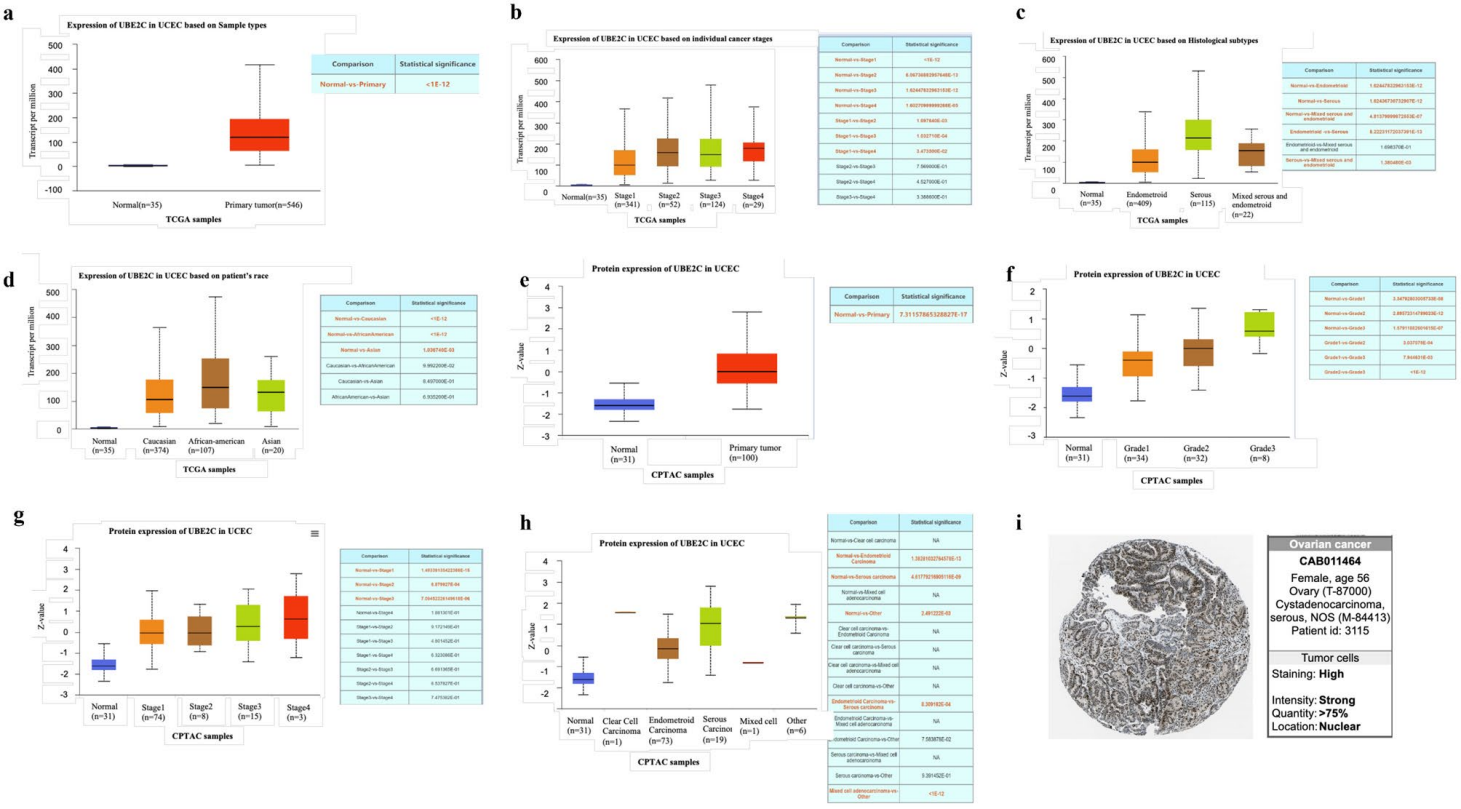
UBE2C mRNA expression and proteomic expression levels in UCEC. Expression of UBE2C based on sample type (**a**), individual cancer stages (**b**), histological subtypes (**c**) and patient’s race (**d**). Box plot and p-value were produced using UALCAN (http://ualcan.path.uab.edu/index.html). The UBE2C proteomic expression profile based on sample types (**e**), tumor grade (**f**), individual cancer stages (**g**) and tumor histology (**h**). **i.** IHC staining of UBE2C was produced from HPA database.

UBE2C mRNA level was validated in normal and UCEC tumor samples applying the UALCAN web tool. UBE2C mRNA level was significantly (p < 0.05) elevated in the UCEC samples compared with the normal tissues (normal n = 35, tumor n = 546) (Fig. 2a). UBE2C showed significantly higher mRNA levels in stage 2, 3, 4 versus stage 1, indicating its potential role in cancer progression (Fig. 2b). UBE2C mRNA level in serous UCEC was significantly higher versus endometroid, or mixed serous and endometroid UCEC, but there was no significant difference among endometroid UCEC, and mixed serous and endometroid UCEC, illustrating that UBE2C mRNA level has its specific role in distinguish serous UCEC from other histological types of UCEC (Fig. 2c). Figure 2d showed that UBE2C was over-expressed in UCEC regardless of patient’s races. Since menopause, age, weight and TP53 status were UCEC risk factors, the association of UBE2C with these factors was further investigated (Supplementary Fig. 1a–d). The expression of UBE2C in post-menopause status was significantly higher versus pre-menopause and peri-menopause status. UBE2C level were similar among different patient’s age and weight groups. But higher mRNA level of UBE2C was observed in TP53-mutant status than in normal and TP53-nonmutant status. The mRNA expression level of UBE2C may act as a supplementary biomarker for molecular classification and high-risk evaluation of UCEC. Moreover, the IHC staining of UBE2C in ovarian carcinoma tissues obtained from the HPA database also showed that UBE2C was highly expressed in ovarian cancer (Fig. 2i).

### Proteomic expression level of UBE2C in UCEC

Using UBE2C as the query gene and UCEC as the CPTAC dataset, the UBE2C proteomic expression profiles based on sample types, individual cancer stages, patient’s age, weight, race, tumor grade, and tumor histology were depicted. Protein level of UBE2C was higher in primary UCEC (n = 100) compared to normal endometrial tissue (n = 31) (Fig. 2e). UBE2C protein level was gradually elevated from grade 1 to grade 3 (Fig. 2f), indicating that UBE2C protein may be a specific biomarker for UCEC cancer cell differentiation. Relative to normal tissues (n = 31), protein level of UBE2C was significantly higher in stage 1 (n = 74), 2 (n = 8) and 3 (n = 5) (Fig. 2g); UBE2C protein was also differentially expressed between normal vs. endometrial carcinoma, normal vs. serous carcinoma, normal vs. others, endometrial carcinoma vs. serous carcinoma, and mixed cell adenocarcinoma vs. others with statistical significance (Fig. 2h). Besides, no significant differences of UBE2C protein level were found among other different groups of tumor stage, histology, patient’s race, age and weight (Fig. 2g and h, Supplementary Fig. 2a–c).

### Survival analysis of UBE2C in UCEC

**Figure 3:**
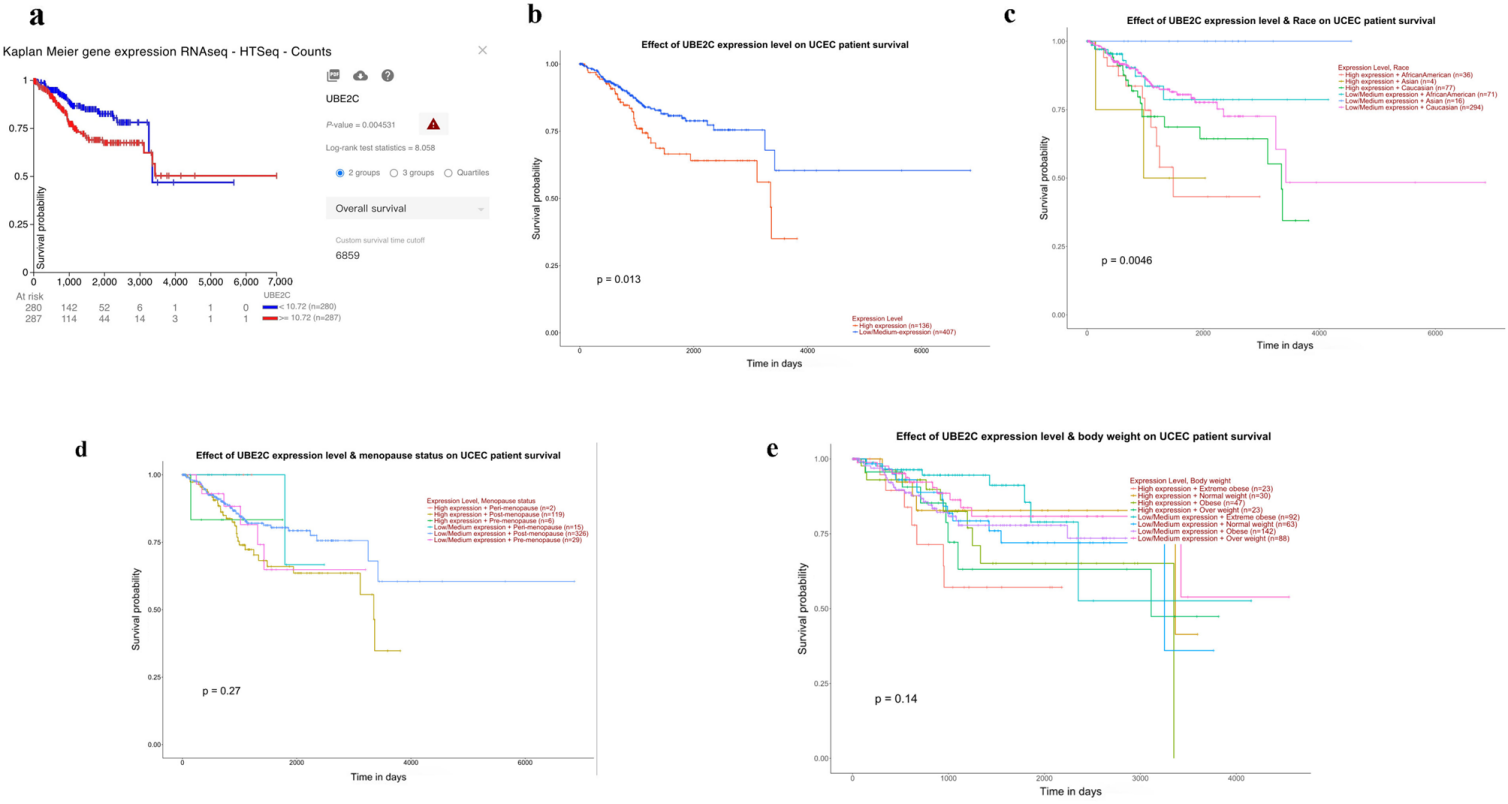
Survival analysis of UBE2C. (**a**) Kaplan Meier-plotter analysis according to UBE2C expression. Effect of UBE2C expression level on patient survival (**b**), & race (**c**), & menopause (**d**) and & body weight (**e**).

The association of UBE2C mRNA level with UCEC prognosis was analyzed using the UCSC Xena and UALCAN web tool. UBE2C’s mRNA level was significantly correlated with the overall survival of UCEC patients. Using 10.72 as UBE2C expression cutoff, patients with higher UBE2C mRNA level (n = 287) showed poorer overall prognosis compared with those with lower UBE2C mRNA level (n = 280) (Fig. 3a). UALCAN showed the similar results that the overall survival was significantly shorter for UCEC patients with higher UBE2C mRNA level (n = 136) compared to those with low-medium UBE2C mRNA level (n = 407) (p = 0.013) (Fig. 3b). UBE2C mRNA level also significantly affected the overall survival of UCEC patients among African American (n = 107), Asian (n = 20) and Caucasian (n = 371) races (p = 0.0046) (Fig. 3c). Further analysis found the overall survivals between African American and Caucasian patients were significantly different in both high and low UBE2C group (p = 0.01, Supplementary Fig. 3). But no significant effects of UBE2C expression level on patient survival were observed among different menopause status and body weight groups (Fig. 3d,e). These results indicated that higher UBE2C mRNA level associated with poorer UCEC patients’ prognosis.

### Identification of key co-expression proteins and protein–protein interaction (PPI) network of UBE2C in UCEC

**Figure 4:**
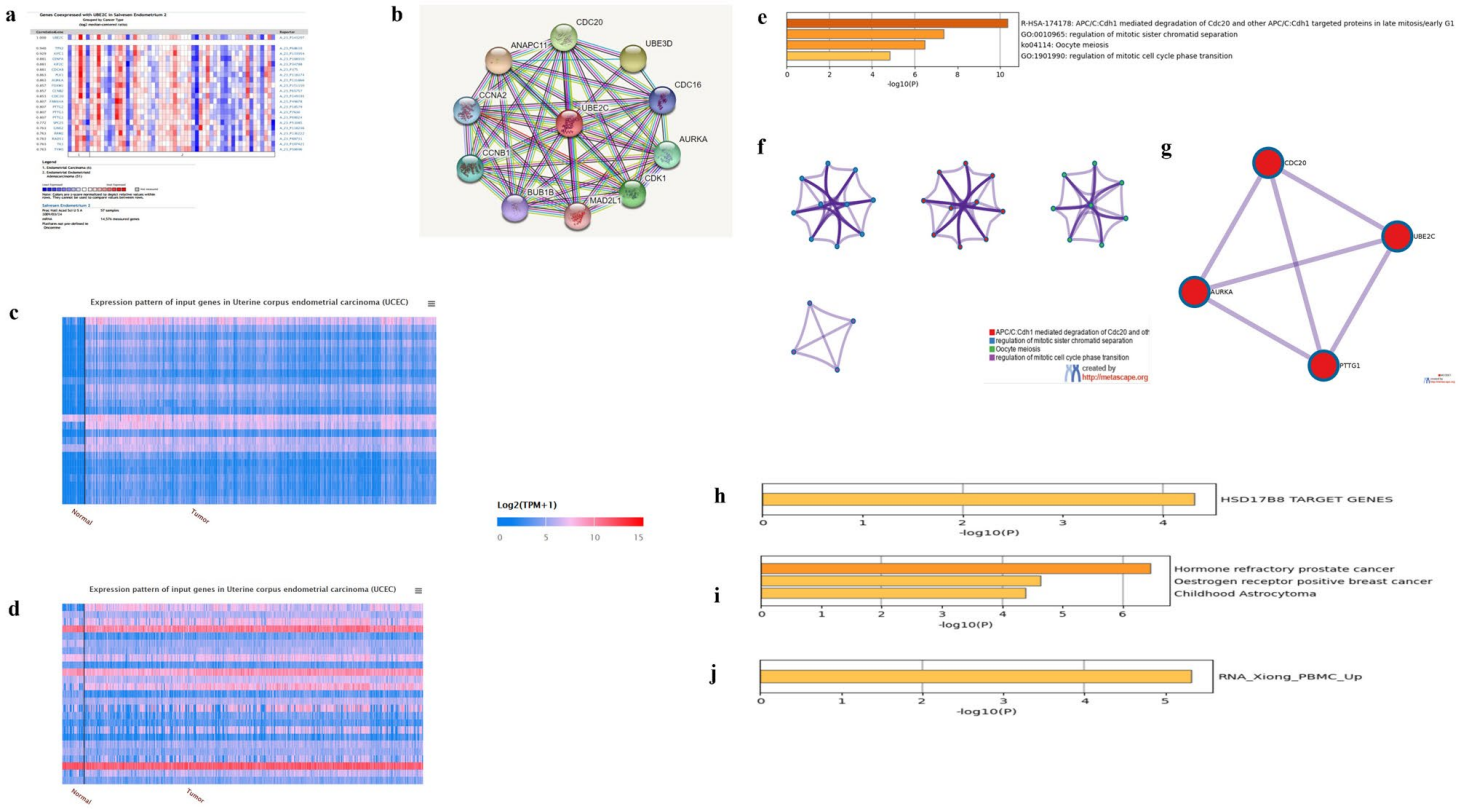
The heatmap and PPI network of key co-expression proteins associated with UBE2C and functional enrichments analysis of UBE2C and key co-expression proteins in Metascape. (**a**) Genes co-expressed with UBE2C by Oncomine. (**b**) PPI network analysis of UBE2C by STRING. (**c**) Positively expression pattern of UBE2C in UALCAN. (**d**) Negatively expression pattern of UBE2C in UALCAN. (**e**) Bar graph, colored by p-values, of enriched terms across input gene lists. (**f**) Network of enriched terms colored by cluster ID. (**g**) Protein–protein interaction network and MCODE components illustrated in the gene lists. (**h**) Summary of enrichment analysis in Transcription_Factor_Targets. (**i**) Summary of enrichment analysis in DisGeNET. (**j**) Summary of enrichment analysis in COVID.

By online platform Oncomine, genes co-expressed with UBE2C were identified and shown in a heatmap (Fig. 4a). The coefficients of genes correlated to UBE2C were showed in the left of the heatmap. The PPI network of UBE2C was established using STRING (Fig. 4b). The genes positively and negatively correlated with UBE2C in UCEC were analyzed using UALCAN and shown in the heatmaps in Fig. 4c and d. Top 20 genes positively and negatively correlated with UBE2C and their Pearson correlated coefficient were listed in Supplementary Tables 2 and 3. The key co-expression proteins which were intersected among Oncomine, STRING and UALCAN were CDC20, AURKA and PTTG1 (Supplementary Table 4). As shown in Supplementary Fig. 4, the expression of CDC20, PTTG1 and AURKA were positively correlated with UBE2C in UCEC regardless of races. These three key genes were significantly elevated in primary tumors relative to normal tissues, and the higher mRNA level, the poorer patient survival (Supplementary Fig. 5).

### Functional enrichments analysis of UBE2C and key co-expression proteins

UBE2C, CDC20, AURKA and PTTG1 were input as a list in Metascape to investigate their potential functional enrichment analysis. Pathway and process enrichment analysis showed that top four clusters with their representative enriched terms (Fig. 4e, Supplementary Table 5). The key genes were participated in APC/C: Cdh1 mediated degradation of Cdc20 and other APC/C: Cdh1 targeted proteins in late mitosis/early G1, regulation of mitotic sister chromatid separation, oocyte meiosis, and regulation of mitotic cell cycle phase transition (Fig. 4f). Protein–protein Interaction Network MCODE Components were shown in Fig. 4g. Quality control and association analysis gene list enrichments were performed in the following ontology categories: Transcription_Factor_Targets, DisGeNET and COVID (Fig. 4h–j).

### Genetic alterations and post translational modifications (PTMs) of UBE2C gene

**Figure 5:**
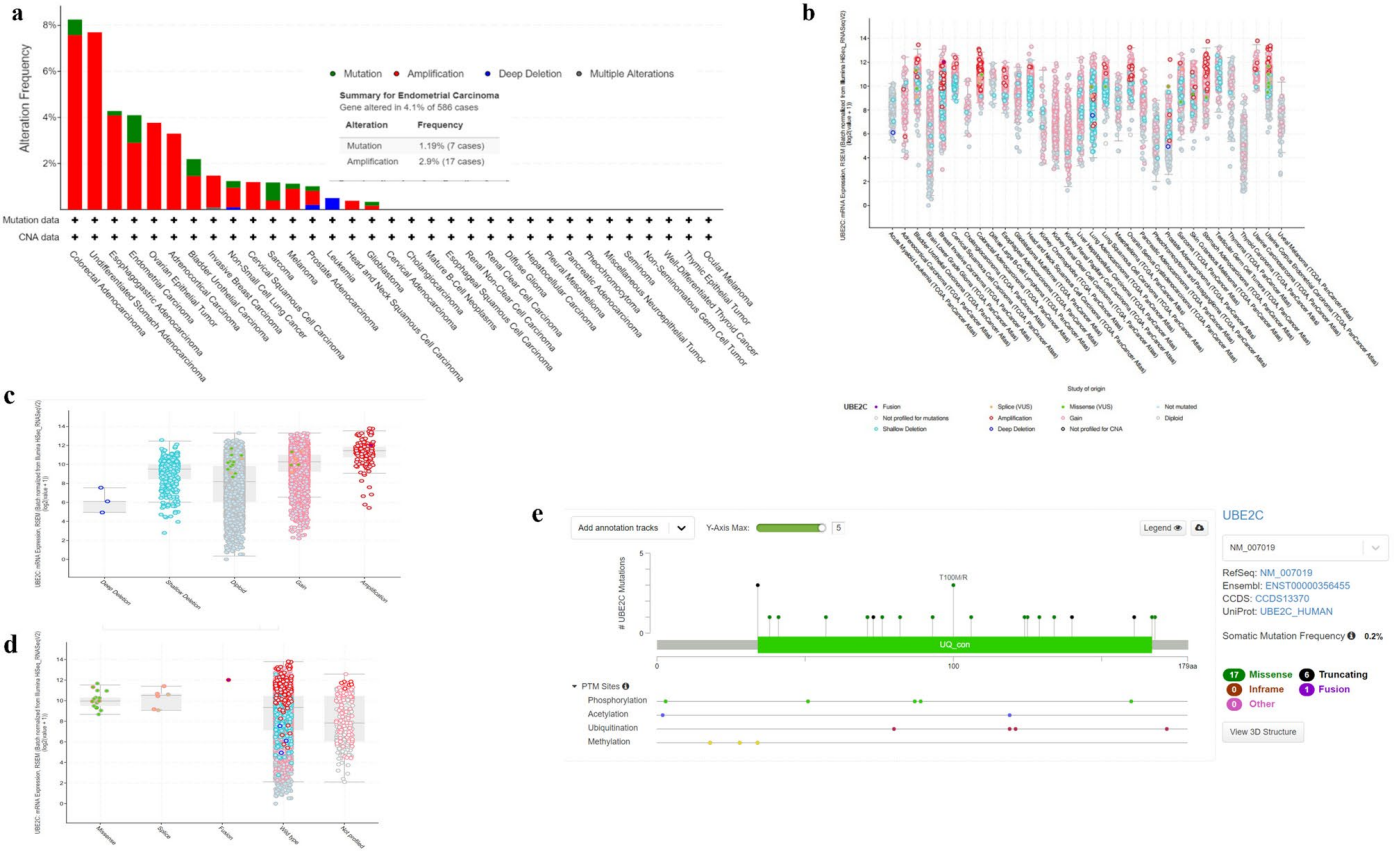
Genetic Alterations of UBE2C. (**a**) Alteration frequency of UBE2C in pan-cancers. (**b**) UBEC mRNA expression from different studies. (**c**,**d**) UBE2C mRNA expression among different gene alteration. (**e**) PTE sites of UBE2C.

The genetic alterations and PTMs of *UBE2C* gene were analyzed in all the different cancers available in the cBioPortal database. There was a total of 32 studies including 10,967 samples analyzed by cBioPortal. The alteration frequency of UBE2C among 33 cancers were shown in Fig. 5a. In most cancers, the most frequently occurred genetic alteration was amplification, then mutation, deep deletion and multiple alterations. In UCEC, gene altered in 4.1% of 586 cases, including 1.19% (7 cases) of mutation and 2.9% (17 cases) of amplification. UBE2C mRNA level from different studies was shown in Fig. 5b. In most studies, the amplification was also the highest alteration of *UBE2C* gene. Figure 5c and d showed that among different gene alteration, amplification and wild type contributed to high level of UBE2C mRNA. The results from genetic alteration analysis indicated that *UBE2C* gene transcription might be partly impacted by genetic alterations. According to bioinformatical prediction analysis, post translational modifications (PTMs) sites such as phosphorylation, acetylation, ubiquitination and methylation might were available for the Ensembl transcript ENST00000356455 (Fig. 5e).

### DNA methylation levels of UBE2C gene in UCEC

Promoter methylation level of *UBE2C* gene in UCEC was performed by UALCAN. *UBE2C* gene promoter methylation profile based on cancer stages, patients’ race, age, weight, tumor grade, histology and TP53 mutation status were also performed by UALCAN (Supplementary Fig. 6). *UBE2C* gene had significantly lower methylation level in primary tumor compared to normal tissue, which was coordinate with over-expression of UBE2C in primary tumor, indicating that hypomethylation of UBE2C might be partly responsible for the overexpression of UBE2C. There was no significant difference in the promoter methylation level of *UBE2C* gene among different groups of tumor stage, patient’s race, age, weight, tumor grade and tumor histology. But the promoter methylation levels of *UBE2C* gene was lower in TP53 mutant tumor samples, indicating that the promoter methylation level of *UBE2C* gene could be applied to distinguish TP53 mutant and non-mutant samples as a diagnostic biomarker.

### UBE2C promoted UCEC cell proliferation, migration and invasion

**Figure 6:**
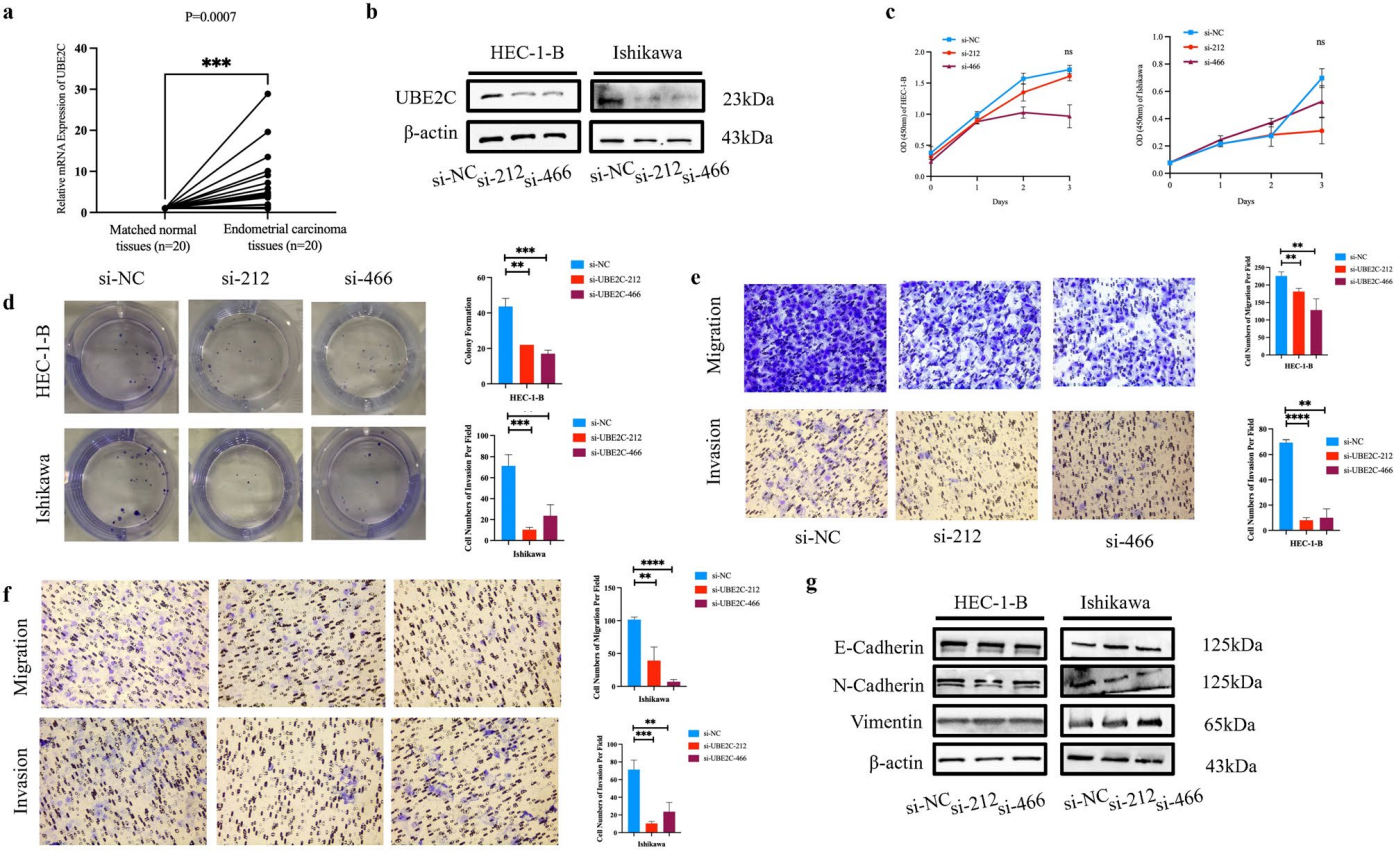
UBE2C was overexpressed in UCEC and promoted UCEC cell malignancy. (**a**) The RT-qPCR of UBE2C between endometrial carcinoma tissues (n = 20) and matched normal tissues (n = 20). (**b**) The Western blot of knocking down UBE2C in HEC-1-B and Ishikawa. (**c**,**d**). The CCK8 assays (**c**) and colony formation (**d**) of knocking down UBE2C in HEC-1-B and Ishikawa. (**e**) The migration and invasion of knocking down UBE2C in HEC-1-B. (**f**) The migration and invasion of knocking down UBE2C in Ishikawa. (**g**) The Western blot of E-Cadherin, N-Cadherin and Vimentin when knocking down UBE2C in HEC-1-B and Ishikawa. The blots have been cropped to improve the conciseness and clarity of the display. The uncropped images were presented in Supplementary Fig. S7.

To verify the bioinformatical results, we performed RT-qPCR to examine UBE2C mRNA level in clinical tissues. Compared with normal adjacent endometrial tissues (n = 20), UBE2C was significantly upregulated in matched endometrial carcinoma tissues (n = 20, Fig. 6a). The relationship between UBE2C overexpression and tumor stages in UCEC suggested the pro-cancer role of UBE2C in UCEC development. To verify this hypothesis, the effect of loss-of-function of UBE2C in the malignancy of UCEC cell lines, HEC-1-B and Ishikawa, was observed. UBE2C knockdown by siRNA transfection (Fig. 6b) significantly retarded cell viability and proliferation as illustrated by CCK8 and plate colony formation assays ((Fig. 6c and d). Knocking down of UBE2C reduced the capabilities of migration and invasion of HEC-1-B cells (Fig. 6e and f). Since epithelial-mesenchymal transition (EMT) is one of the most important molecular events promoting cancer cells migration and invasion, the association between UBE2C and EMT was then investigated. Western blotting results showed that the expression of classic epithelial marker (E-cadherin) and mesenchymal markers (N-cadherin and vimentin) remained largely unchanged when UBE2C was downregulated (Fig. 6g), indicating that UBE2C might promote UCEC progression through other molecular pathways. The blots have been cropped to improve the conciseness and clarity of the display. The uncropped blot images were presented in Supplementary Fig. S7. In all, these results indicated that UBE2C was a pro-tumor molecule in UCEC.

## Discussion

UCEC is one of the most common gynecological tumors, with high risk factors such as menopause status and TP53 mutation. The five- year survival rate for advanced UCEC is only 16%. Therefore, it is of great importance and urgency to discover new reliable clinical detection biomarkers for early diagnosis, effect treatment and prognostic prediction. Integrated bioinformatics analysis is a cost-effective way to identify effective biomarkers. In this study, UBE2 family and specifically UBE2C was analyzed in UCEC using bioinformation tools. The study results provided a comprehensive insight of clinical significance and oncogenic role of UBE2C.

UBE2C, also known as UBCH10, establishes the topology of ubiquitin chains and takes part in cell cycle progression by specifically interacting with the anaphase-promoting complex/cyclostome (APC/C) to determine the consequences of ubiquitylation for the modified proteins^23,24^. A series of studies have demonstrated that UBE2C is overexpressed in various human malignancies^25–27^. Increasing evidence has revealed the importance of UBE2C as a potential candidate proto-oncogene functioning in tumorigenesis, tumor progression, epithelial-mesenchymal transitions (EMT), autophagy and cisplatin-based chemotherapeutic resistance^26,28–32^. Estradiol induced estrogen receptor α to enhance UBE2C transcription and thus promoted endometrial cancer cell proliferation and EMT via increasing p53 ubiquitination and degradation^31^. However, the comprehensive bioinformatical analysis, the function and the underlying regulatory mechanisms of UBE2C in UCEC remain to be elucidated. Of course, bioinformatical analysis approaches have their unique advantages and several limitations as well. For example, big data analysis means to integrate multiple different datasets from different labs, where maybe using different chip assays, different analytical approaches and different information collection, which causes bias and needs to be batched and normalized for further analysis. And due to the discordant economic development level and emphasis on medical among developed and undeveloped countries, the data from Caucasian is currently the most integrated. Other races especially African-American and Asian’ data are urgently needed to be completed and optimized.

By analyzing the differentially expressed genes in UCEC compared with normal tissues, nine specific UBE2 family genes possibly related to UCEC have been screened out. With the combination of mRNA level analysis and survival prognosis among these nine genes, UBE2C was selected as the most significantly over-expressed gene with clinical prognosis value. Its expression level was correlated with cancer stages, histological subtypes and poor prognosis, indicating its oncogenic role in cancer development and as a potential biomarker for cancer molecular diagnosis and prognosis prediction. Also, the expression of UBE2C was correlated with patient’s menopause statue and TP53 mutation status, in accordance with the basic characteristic of UCEC. Genetic alterations such as mutation, amplification and promoter hypomethylation may all contribute to its high expression. Furthermore, in vitro experiments demonstrated that UBE2C stimulated UCEC cell migration and invasion without influencing EMT process. There may be other underlying potential molecular mechanism of UBE2C in functioning cell migration and invasion. Due to its role in promoting cancer migration and invasion, UBE2C could be an effective biomarker for UCEC metastatic prediction and monitor.

During the comprehensive bioinformatics analysis, we also dig out three key co-expression proteins positively correlated with UBE2C. These three key molecules, CDC20, PTTG1 and AURKA, were highly expressed in UCEC compared with normal samples and their expression levels were associated with poor prognosis. Recent studies have demonstrated that UCEC patients with down-regulated CDC20 showed better overall survival, and UCEC cell proliferation was suppressed by knocking down CDC20^33^. Overexpression of PTTG1 may participate in pathogenesis and progression of UCEC. PTTG1 may act as an effective biomarker for UCEC diagnosis and prognosis prediction^34,35^. Overexpression of AURKA was an unfavorable prognostic factor in endometrioid ovarian cancer and UCEC^36,37^. However, the interaction and detailed regulation among these proteins were unclear, further researches needed to be done to reveal their underlying connections.

Therefore, based on the comprehensive bioinformatical analysis and molecular experiments, we found that UBE2C was highly expressed and could serve as oncogenic molecule in UCEC progression and development. UBE2C could be an effect biomarker for cancer prognosis prediction and metastasis monitor.

## Materials and methods

### mRNA expression and prognosis analysis of UBE2 family members in UCEC

The online databases, Gene Expression Profiling Interactive Analysis (GEPIA, http://gepia.cancer-pku.cn/)^38^ and GEPIA 2 (http://gepia2.cancer-pku.cn/#general)^39^ were used to screen the differential expression genes of UBE2 family between UCEC and normal endometrial tissues. In addition, the databases were used to investigate the prognosis value of the selected UBE2 family members in UCEC. p < 0.05 indicated a difference with statistical significance.

### mRNA expression analysis of UBE2C in UCEC

UALCAN (http://ualcan.path.uab.edu/index.html)^40^ is an online web tool that can be applied to analyze tumor transcriptome data. RNA level of UBE2C in tumor and the compared normal samples of UCEC was validated and further investigated by using the UALCAN web tool based on TCGA datasets. Expression of UBE2C in UCEC based on patient’s race, weight, age, menopause status, histologic subtypes, tumor stages and TP53 mutation status were also performed by UALCAN. Statistical significance was computationally calculated. The Human Protein Atlas (https://www.proteinatlas.org/) was used to explore the protein localization and expression of UBE2C in UCEC tissues.

### Proteomic expression analysis of UBE2C in UCEC

Protein expression level of UBE2C in UCEC based on patient’s age, weight, race, sample types, tumor stages, grade and histology were performed by UALCAN based on CPTAC analysis. Z-values was represented standard median deviations across the given cancer type samples.

### Survival analysis of UBE2C in UCEC

Effect of UBE2C expression levels on the survival of UCEC patient was performed by UCSC Xena (http://xena.ucsc.edu/)^41^ and UALCAN. Effect of UBE2C expression levels, races, menopause status and body weight on the survival of UCEC patient were also performed by UALCAN. For the effect of UBE2C expression level and race, combined survival plot and individual survival plots were investigated, respectively. p values were calculated automatically.

### Identification of key co-expression proteins and protein–protein interaction (PPI) network of UBE2C in UCEC

The co-expression profiles of UBE2C in UCEC were obtained from Oncomine and were explained as a heat map. Search Tool for the Retrieval of Interacting Genes (STRING) (http://string-db.org/)^42^, was using UBE2C as a query to predict its potential interactive proteins. The output was illustrated as a network showing the relationships between genes, in which nodes represented genes and links symbolized networks at a 0.90 confidence level. Genes positively and negatively correlated with UBE2C in UCEC were analyzed by UALCAN. Genes with extremely low expression (Median TPM < 0.5) were filtered out of the list. A heat map was applied to represent the co-expression profiles of UBE2C in UCEC. Pearson correlation coefficient was calculated and ranked to perform its positively and negatively correlation proteins. Key co-expression proteins of UBE2C were identified as the intersection outcome among STRING, Oncomine and UALCAN. Expression correlation plots, GEx profiles and survival profiles of key genes were investigated by UALCAN.

### Functional enrichments analysis of UBE2C and key co-expression proteins

A list of UBE2C and key co-expression proteins were imputed into Metascape (https://metascape.org/gp/index.html#/main/step1)^43^, a Gene Annotation & Analysis Resource, for pathway and process enrichment analysis, protein–protein interaction enrichment analysis, quality control and association analysis.

### Genetic alterations analysis of UBE2C gene

Genetic alterations of *UBE2C* gene were performed by cBioPortal, an online open access platform for exploring cancer genomics (http://www.cbioportal.org/)^44^. TCGA PanCancer Atlas Studies were selected for visualization and analysis. Mutation and copy number alterations were selected as molecular profiles. *UBE2C* was input as query gene.

### DNA methylation analysis of UBE2C gene in UCEC

Promoter methylation level of *UBE2C* gene in UCEC was performed by UALCAN. *UBE2C* promoter methylation profile based on patients’ race, age, weight, tumor stages, tumor grade, histology and TP53 mutation status were also performed by UALCAN.

### Cell culture

UCEC cell lines HEC-1-B and Ishikawa were from ATCC and cultivated in RPMI-1640 medium supplemented with 10% FBS (HyClone), at 37 °C in humidified 5% CO_2_ atmosphere. All experiments mentioned below were performed in accordance with relevant guidelines and regulations.

### Clinical specimens

A total of 20 freshly frozen normal adjacent tissues and matched endometrial carcinoma tissues were collected from The Department of Obstetrics and Gynecology, Shaanxi Provincial People's Hospital (Shaanxi, China). All patients were diagnosed with endometrial carcinoma without any antitumor therapy before surgery. Detailed clinicopathological information of all patients were collected (Supplementary Table 1). This study was approved by the Ethics Committee of The Shaanxi Provincial People's Hospital and conducted in compliance with the principles of the Declaration of Helsinki for medical research involving humans.

### siRNA knockdown and transient transfection

UBE2C small interfering RNA (siRNA) were bought from GenePharm (Shanghai, China). The UBE2C-siRNA sequences were: siUBE2C-212, 5′-GUCUGGCGAUAAAGGGAUUTT-3′; and siUBE2C-466, 5′-GGACCAUUCUGCUCUCCAUTT-3′. Cells were transfected with the UBE2C siRNA with Lipofectamine 2000 (Invitrogen). 48–72 h after transfection, the knockdown efficiency was determined by RT-qPCR and Western blot. The siRNA-transfected cells were harvested for Transwell migration and invasion assays after 48 h-transfection.

### RT-qPCR analysis

Total RNA was extracted from cells using Trizol reagent (Invitrogen) and then reversely transcribed into cDNA using a reverse transcription kit (Takara; Dalian, China). After that, the mRNA level of UBE2C was detected using the SYBR green premix (Takara). The primers were synthesized by Beijing Genomics Institute (Beijing, China), and the primer sequences were listed below: UBE2C forward: 5’-CGAGTTCCTGTCTCTCTGCC-3’; UBE2C reverse: 5’-CAGCTCCTGCTGTAGCCTTT-3’; β-actin forward: 5’-TCCCTGGAGAAGAGCTACGA-3’; β-actin reverse: 5’-AGCACTGTGTTGGCGTACAG-3’. β-actin was used as an internal control to calculate the expression of UBE2C. The relative expression of UBE2C was automatically calculated using 2^−ΔΔCt^ method by a CFX96 real-time PCR system (Bio-Rad).

### Western blot analysis

Endometrial cancer cells with indicated treatments were lysed using RIPA with 1 mmol/L PMSF at 4 °C for 30 min. The cells were subsequently collected by scraping, followed by centrifugation at 4 °C, 12,000 rpm for 5 min. The supernatant was harvested. Protein samples were fractionated by SDS-PAGE, transferred onto a NC membrane (Pall) followed by incubation overnight at 4 °C with primary antibodies specific to UBE2C (1:1,000, Abcam), E-cadherin (1:500, Cell Signaling Technology), N-cadherin (1:500, Cell Signaling Technology), Vimentin (1:1,000, Cell Signaling Technology), or β-actin (1:2,000, Cell Signaling Technology). After incubation with horseradish peroxidase (HRP)-conjugated secondary antibodies (1:1,000), immune complexes were detected using enhanced chemiluminescence detection reagents (Thermo Fisher Scientific) on Image Lab Software in Molecular Imager ChemiDoc XRS (Bio-Rad).

### CCK8 assays

Cells were seeded at a density of 10^3^ cells/well into 96-well plates and cultured in a 37 °C incubator. Cells were transfected with UBE2C-specific siRNAs or negative control siRNA. 48 h after transfection, Cell Counting Kit-8 (CCK-8, 7 sea pharmtech Co., Ltd.; Shanghai, China) was applied to measure the relative cell viability based on the manufacturer's protocol. Each sample was measured at 450 nm as an optical density (OD) value. The experiments were conducted in triplicates and expressed as mean ± SD (*p < 0.05).

### Colony formation

400 cells/well were seeded into 12-well plates and cultured for 2 weeks in an incubator containing 5% CO_2_ at 37 °C until colony formation. Then, the cell colonies were washed three times with PBS and fixed with methyl alcohol, stained with 0.1% crystal violet. Colonies were counted as the colony containing more than 50 cells. The experiments were conducted in triplicates and expressed as mean ± SD (*p < 0.05).

### Transwell cell migration and invasion assays

Transwell assays were performed using a 24-well plate containing the lower and upper chambers partitioned by a polycarbonate membrane (8-μm pore size). Cells (3–5 × 10^5^/well) were seeded in RPMI-1640 without FBS in the top chamber, and the bottom chambers contained RPMI-1640 with 20% FBS. Cells were allowed to migrate or invade for 24 to 48 h in an incubator containing 5% CO_2_ at 37 °C. Cells stayed on the upper side of the membrane were removed using cotton swabs. The membrane was then washed by PBS for three times and fixed in methyl alcohol for 30 min and stained with crystal violet. Cells that passed the membrane were imaged and counted in five independent fields. For the invasion assay, Matrigel (BD Biosciences) was applied to the top chambers before following the migration assay method. Each experiment was performed three times.

### Statistical analysis

Statistical analyses were performed using GraphPad Prism version 9 (GraphPad Software, La Jolla, CA, USA). Survival curves were plotted using the data extracted from the PrognoScan and Kaplan–Meier plotters. All results were displayed with p-values. Student's t test and one-way ANOVA were used for two- and multi-group comparisons, respectively. P-value < 0.05 was considered to be statistically significant.

### Supplementary Information

Supplementary Information.
