# Supplementary material for: UBE2C serves as a prognosis biomarker of uterine corpus endometrial carcinoma via promoting tumor migration and invasion
Source: Sci Rep. 2023 Oct 6;13:16899. doi: 10.1038/s41598-023-44189-1 (PMC10558470; doi:10.1038/s41598-023-44189-1)
Supplement: Supplementary file 1 — Supplementary Information. [file 41598_2023_44189_MOESM1_ESM.docx]

Supplementary Material

UBE2C serves as a prognosis biomarker of uterine corpus endometrial carcinoma via promoting tumor migration and invasion

**Sijia Ma ^1,2,3^, Qian Chen ^4^, Xu Li ^1,2,3^, Jing Fu ^1,2,3,^****^*^, Le Zhao ^1,2,3^**

^1^Center for Translational Medicine, the First Affiliated Hospital of Xi’an Jiaotong University, Xi’an, Shaanxi 710061, P.R. China

^2^Department of Obstetrics and Gynecology, the First Affiliated Hospital of Xi’an Jiaotong University, Xi’an, Shaanxi 710061, P.R. China

^3^Key Laboratory for Tumor Precision Medicine of Shaanxi Province, the First Affiliated Hospital of Xi’an Jiaotong University, Xi’an, Shaanxi 710061, P.R. China

^4^Department of Obstetrics and Gynecology, the Second Affiliated Hospital of Xi’an Jiaotong University, Xi’an, Shaanxi 710061, P.R. China

*** Correspondence:**Jing Fu**^*^**
fujing200409@xjtufh.edu.cn

Table S1. ﻿Clinicopathological characteristics of collected clinical specimens.

| **Number** | **Age** | **Stage** | **T** | **N** | **M** |
| --- | --- | --- | --- | --- | --- |
| 1 | 52 | Ⅰa | 1a | 0 | x |
| 2 | 47 | Ⅱ | 2 | 0 | x |
| 3 | 71 | Ⅰa | 1a | 0 | x |
| 4 | 49 | Ⅲc1 | 3 | 1 | x |
| 5 | 56 | Ⅲ | 3 | 0 | x |
| 6 | 66 | Ⅰa | 2a | 0 | x |
| 7 | 54 | 1b | 1b1 | 0 | x |
| 8 | 60 | Ⅰb | 1b | 0 | x |
| 9 | 56 | Ⅰb | 1b | 0 | x |
| 10 | 45 | Ⅱ | 2 | 0 | x |
| 11 | 59 | Ⅰa | 1a | 0 | x |
| 12 | 41 | Ⅰb | Ⅰb | 0 | x |
| 13 | 59 | Ⅰa | 1a | 0 | x |
| 14 | 60 | Ⅰa | 1a | 0 | x |
| 15 | 48 | Ⅰa | 1a | 0 | x |
| 16 | 52 | Ⅰa | 1a | 0 | x |
| 17 | 59 | Ⅰa | 1a | 0 | x |
| 18 | 45 | Ⅰa | 1a | 0 | x |
| 19 | 69 | Ⅰa | 1a | 0 | x |
| 20 | 57 | Ⅰa | 1a | 0 | x |

Table S2. Top 20 Genes positively correlated with UBE2C in UCEC.

| Genes | PearsonCC |
| --- | --- |
| TROAP | 0.76 |
| CENPA | 0.73 |
| TPX2 | 0.71 |
| AURKA | 0.71 |
| HJURP | 0.7 |
| KIF2C | 0.69 |
| CDC25C | 0.68 |
| DSN1 | 0.66 |
| MYBL2 | 0.66 |
| CDCA3 | 0.66 |
| KIF20A | 0.66 |
| PIF1 | 0.66 |
| HMGB2 | 0.65 |
| PTTG1 | 0.65 |
| SPC25 | 0.65 |
| TACC3 | 0.64 |
| CSE1L | 0.63 |
| NUF2 | 0.63 |
| FAM54A | 0.63 |
| FAM72B | 0.63 |

Table S3. Top 20 Genes negatively correlated with UBE2C in UCEC.

| Genes | PearsonCC |
| --- | --- |
| TOM1 | -0.47 |
| PPAP2C | -0.42 |
| EEF2 | -0.41 |
| ZDHHC1 | -0.4 |
| SPRYD3 | -0.4 |
| P2RX4 | -0.4 |
| NPDC1 | -0.4 |
| LMF1 | -0.38 |
| EEF1A1P9 | -0.37 |
| SCAMP4 | -0.37 |
| SERINC2 | -0.37 |
| KCNK6 | -0.37 |
| SGSM3 | -0.36 |
| KIAA1324 | -0.36 |
| SORBS2 | -0.36 |
| PEX11G | -0.36 |
| SPDEF | -0.36 |
| C9orf152 | -0.35 |
| ARSD | -0.35 |
| RARA | -0.34 |

Table S4. Key co-expression proteins of UBE2C

| Key Genes | Score（STRING） | Correlation  (Oncomine) | PearsonCC (UALCAN) |
| --- | --- | --- | --- |
| CDC20 | 0.999 | 0.853 | 0.590 |
| AURKA | 0.998 | 0.863 | 0.710 |
| PTTG1 | 0.997 | 0.807 | 0.650 |

Table S5. Top 4 clusters with their representative enriched terms (one per cluster).

| GO | Category | Description | Count | % | Log10(P) | Log10(q) |
| --- | --- | --- | --- | --- | --- | --- |
| R-HSA-174178 | Reactome Gene Sets | APC/C:Cdh1 mediated degradation of Cdc20 and other APC/C:Cdh1 targeted proteins in late mitosis/early G1 | 4 | 100 | -10.36 | -6.29 |
| GO:0010965 | GO Biological Processes | regulation of mitotic sister chromatid separation | 3 | 75 | -7.35 | -3.99 |
| ko04114 | KEGG Pathway | Oocyte meiosis | 3 | 75 | -6.48 | -3.57 |
| GO:1901990 | GO Biological Processes | regulation of mitotic cell cycle phase transition | 3 | 75 | -4.83 | -2.15 |


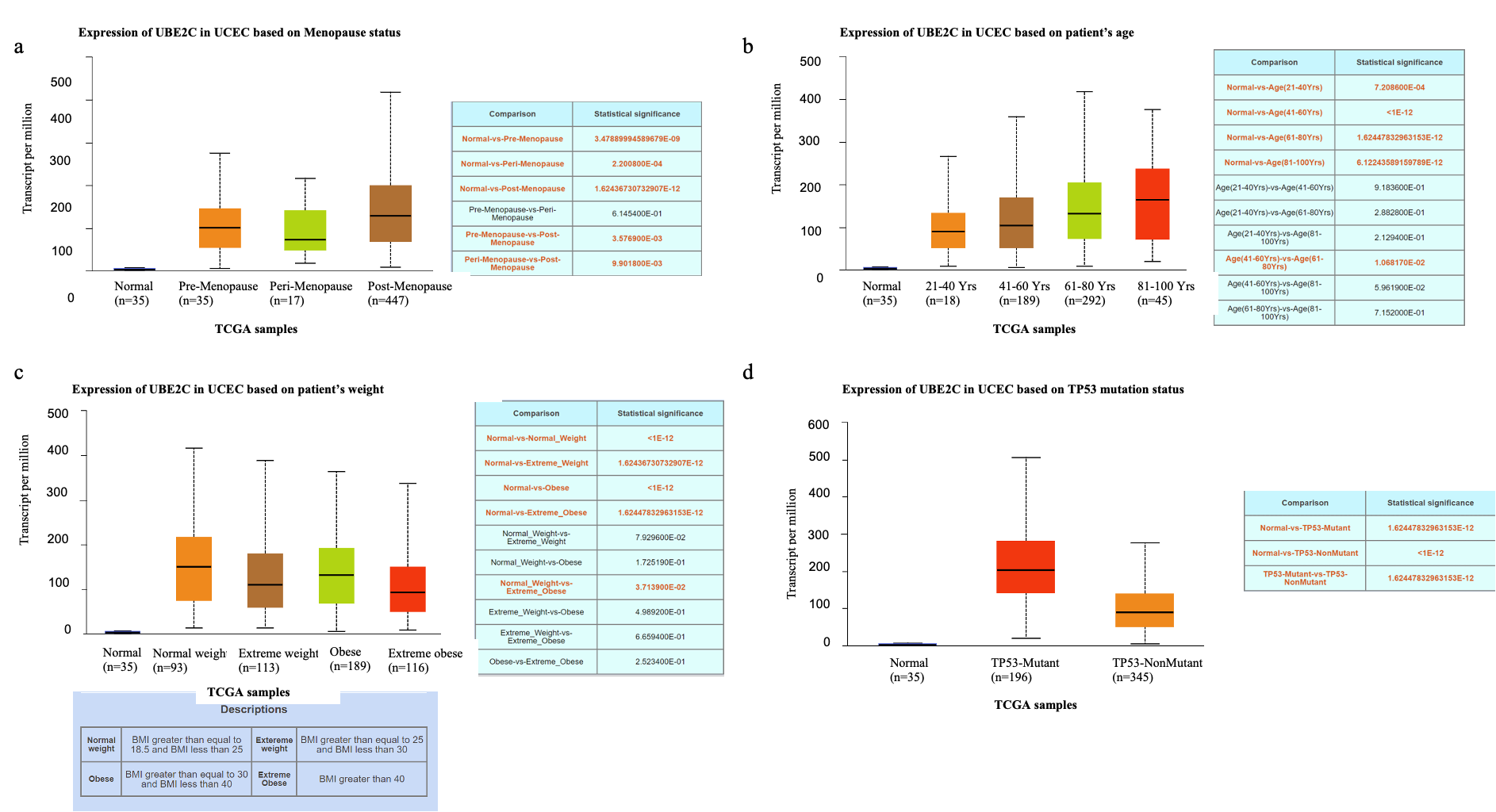


Figure S1. Effect of UBE2C expression level and menopause status (a), patient’s age (b), patient’s weight (c) and TP53 mutation status (d) in UCEC.


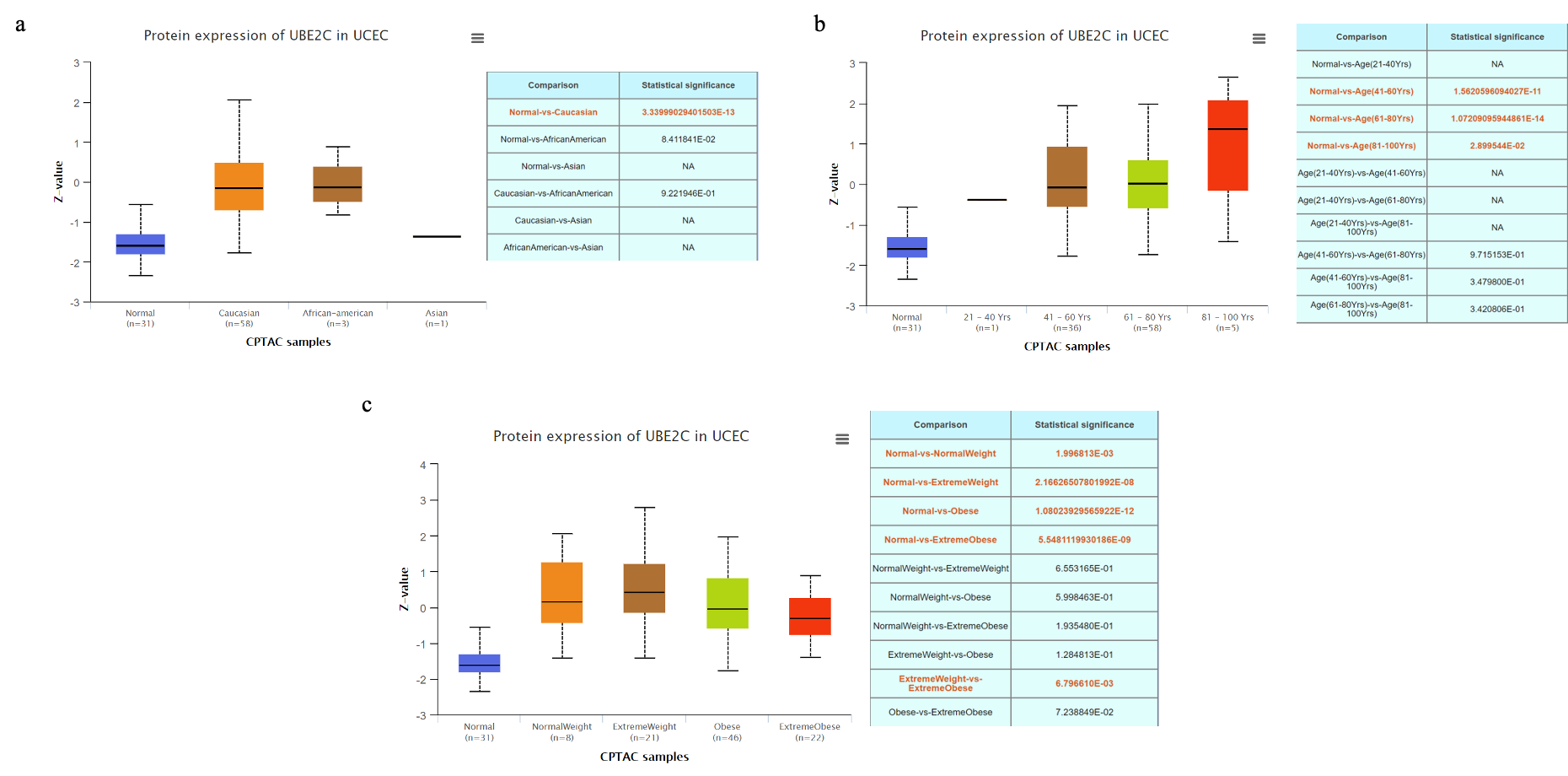


Figure S2. The UBE2C proteomic expression profile based on patient’s race (a), patient’s age (b) and patient’s weight (c).


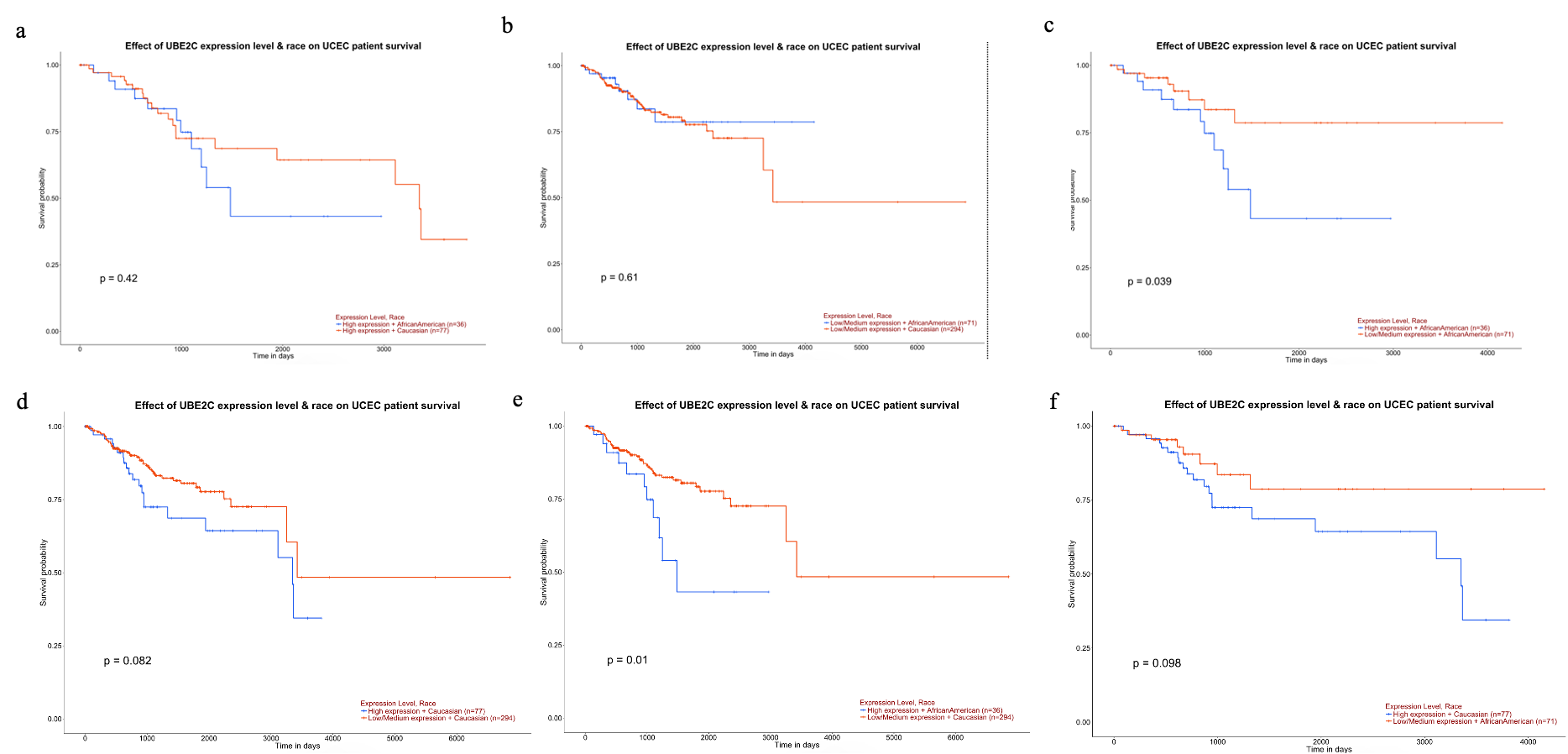


Figure S3. Individual analysis of the effect of UBE2C expression & specific race on the survival prognosis in UCEC. a. The patient survival between high expression plus African American and high expression plus Caucasian. b. The patient survival between low/medium expression plus African American and low/medium expression plus Caucasian. c. The patient survival between high expression plus African American and low/medium expression plus African American. d. The patient survival between high expression plus Caucasian and low/medium expression plus Caucasian. e. The patient survival between high expression plus African American and low/medium expression plus Caucasian. f. The patient survival between high expression plus Caucasian and low/medium expression plus African American.


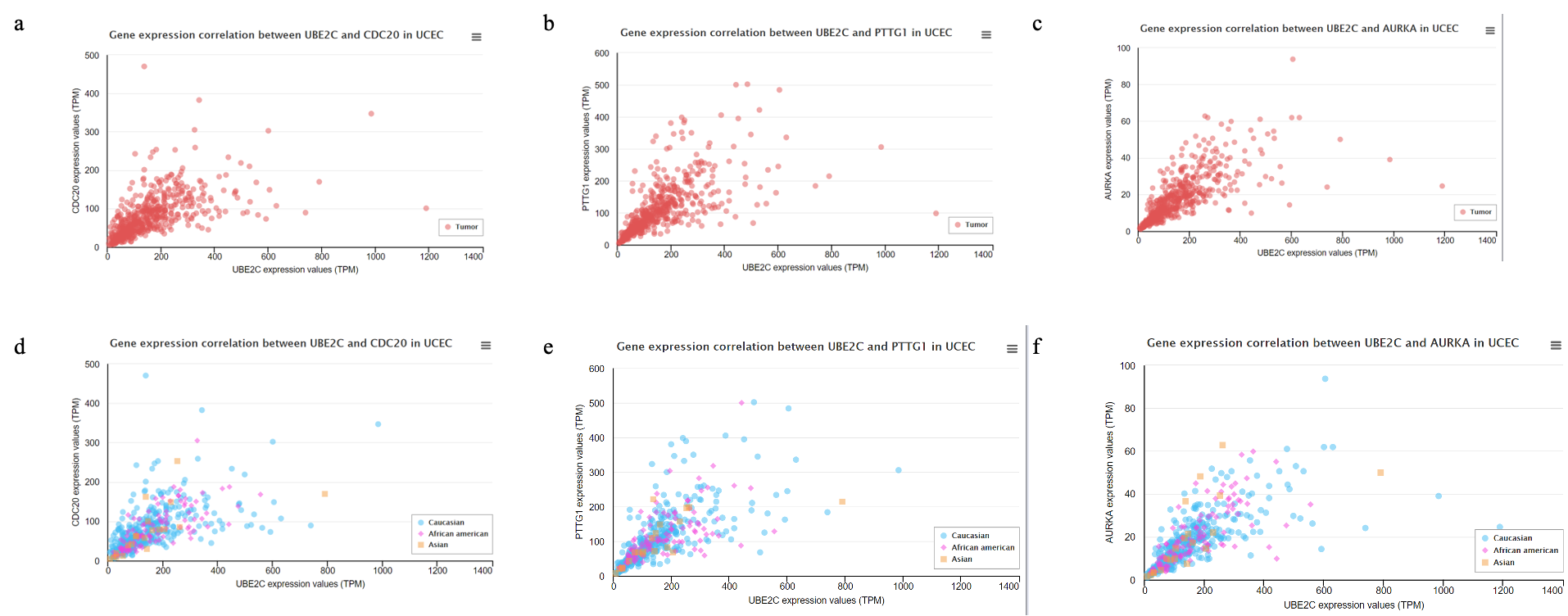


Figure S4. Gene expression correlation among UBE2C and key co-expression genes. Among tumors, gene expression correlation between UBE2C and CDC20 (a), PTTG1 (b), AURKA (c) in UCEC. Among Caucasian, African American and Asian, the gene expression correlation between UBE2C and CDC20 (d), PTTG1 (e), AURKA (f) in UCEC.


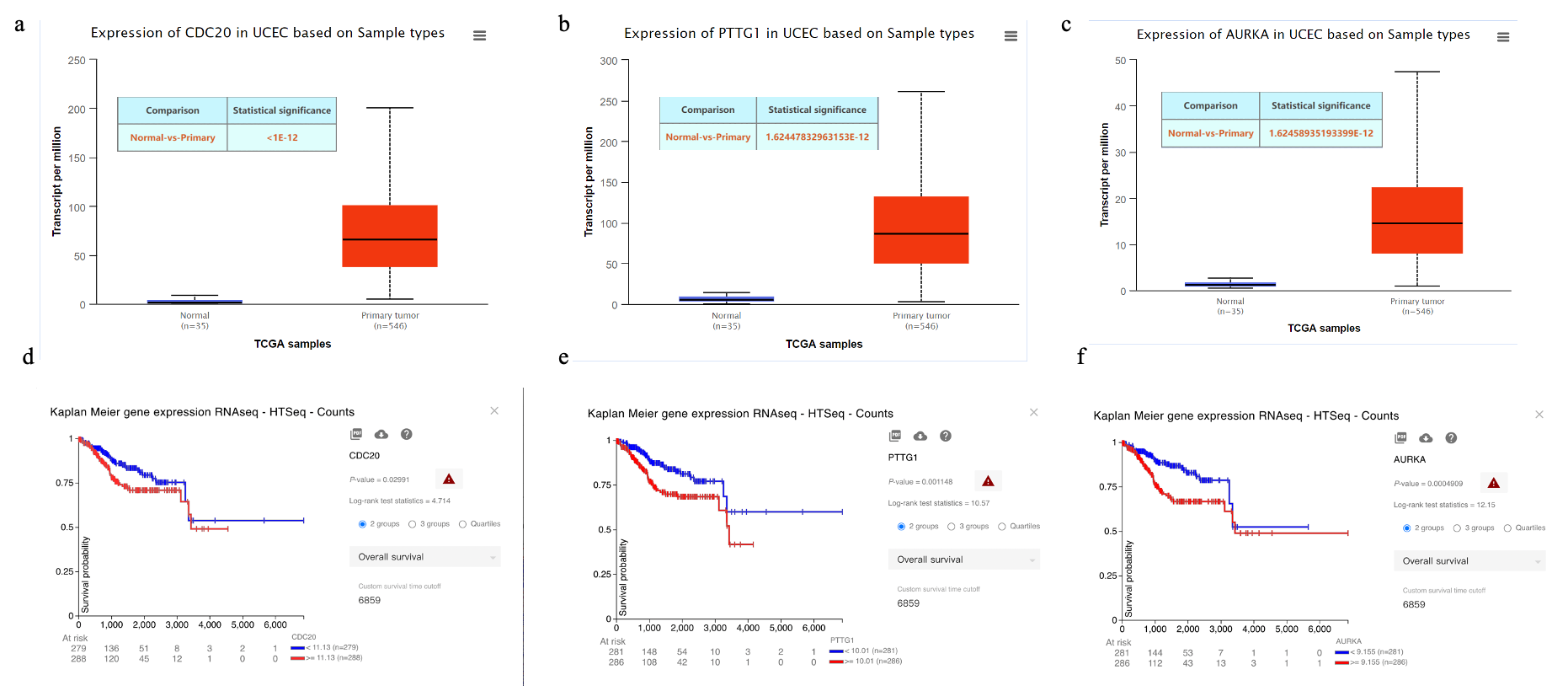


Figure S5. Expression level and Kaplan Meier survival analysis of three key co-expression proteins in UCEC. a. Expression of CDC20 in UCEC based on sample types. b. Expression of PTTG1 in UCEC based on sample types. c. Expression of AURKA in UCEC based on sample types. d. Kaplan Meier plotter of gene CDC20. e. Kaplan Meier plotter of gene PTTG1. f. Kaplan Meier plotter of gene AURKA.


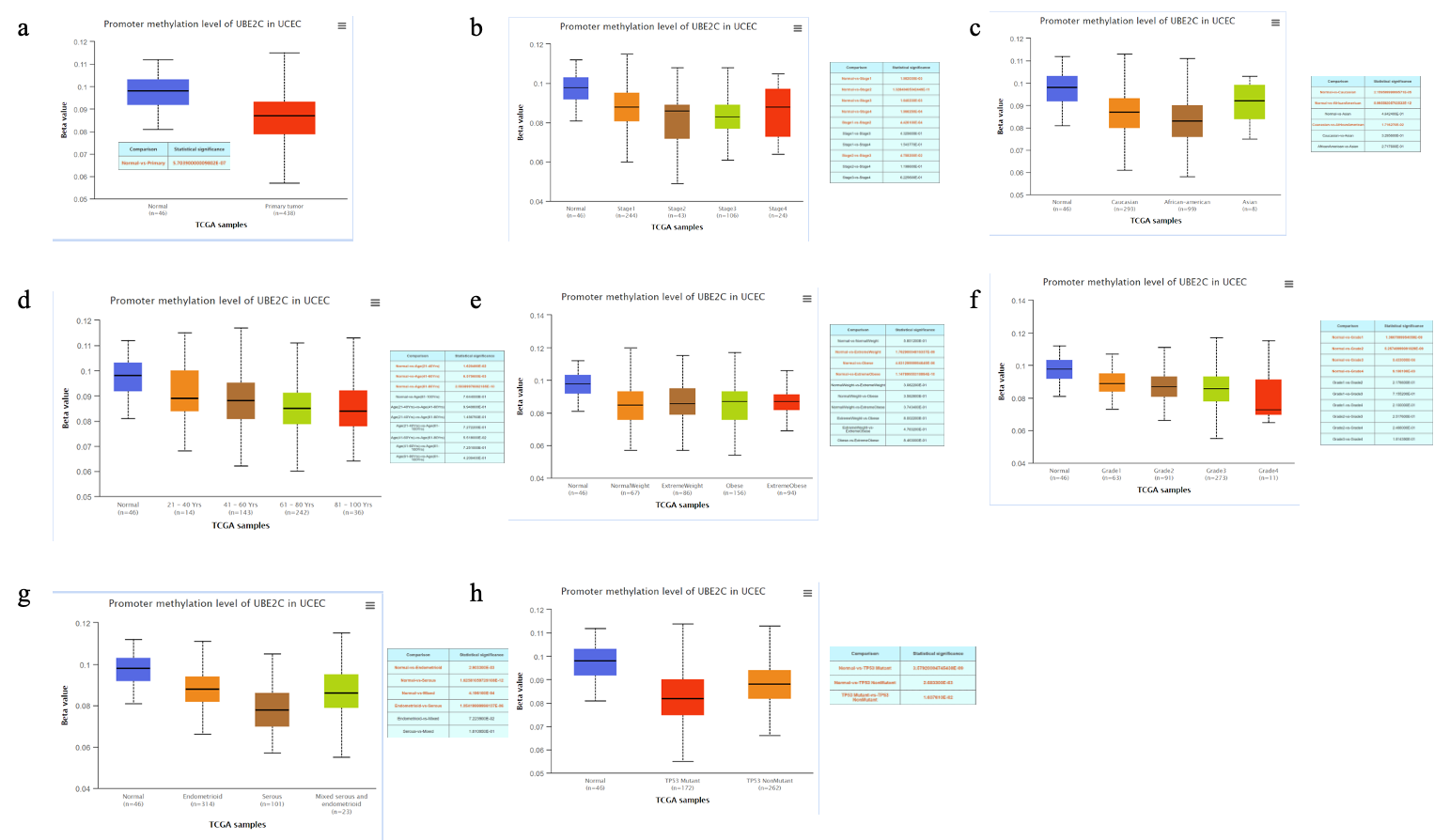


Figure S6. DNA Methylation levels of UBE2C in UCEC. Promoter methylation level of UBE2C in UCEC (a) and UBE2C promoter methylation profile based on cancer stages (b), patients’ race (c), age (d), weight (e), tumor grade (f), histology (g) and TP53 mutation status (h).


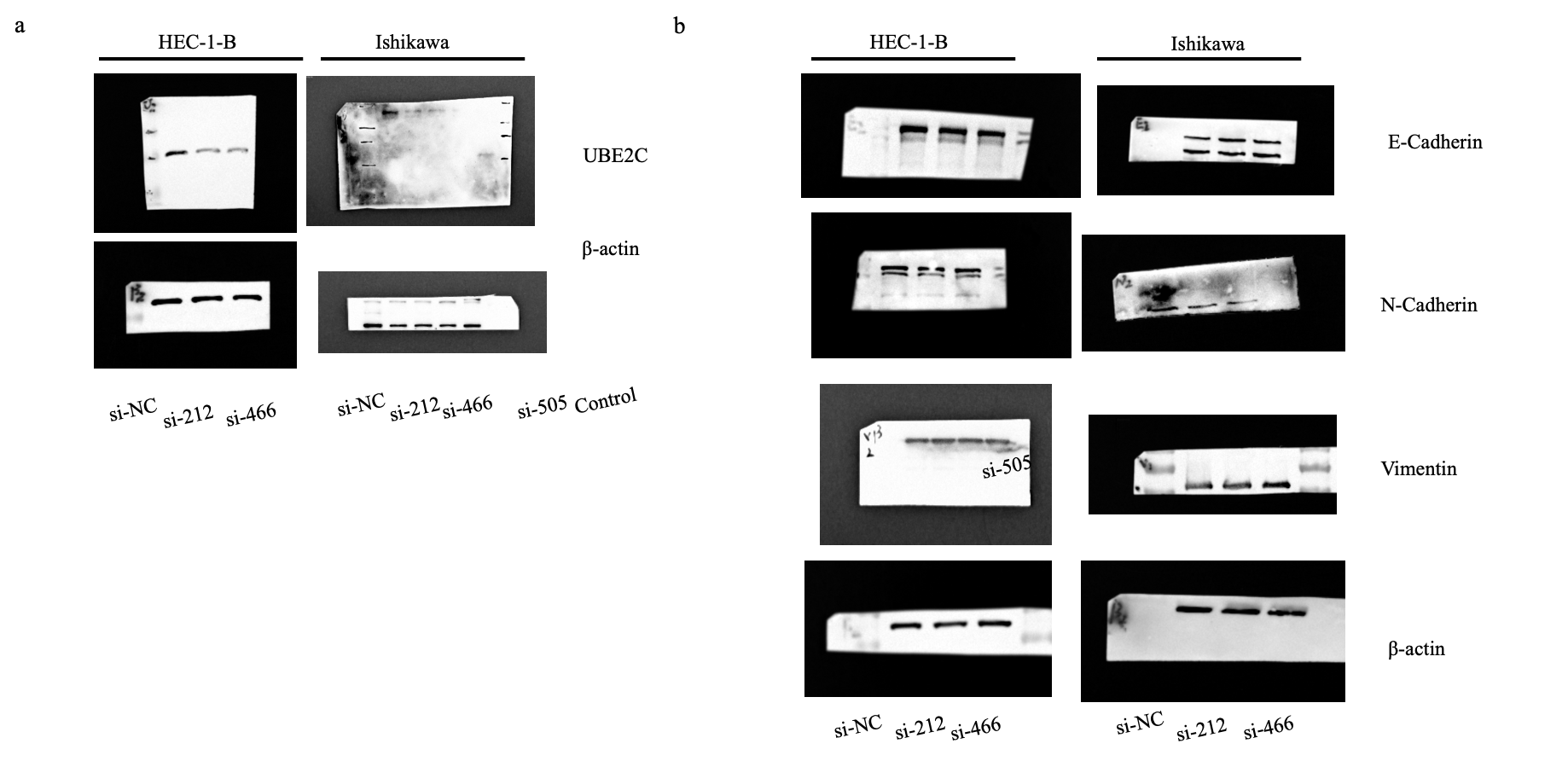


Figure S7. The uncropped images of Figure 7. a. The western blot of knocking down UBE2C in HEC-1-B and Ishikawa. Si-505 was the non-effective UBE2C si-RNA. b. The western blot of E-Cadherin, N-Cadherin and Vimentin when knocking down UBE2C in HEC-1-B and Ishikawa.
